# Differential Survival of Hyper-Aerotolerant *Campylobacter jejuni* under Different Gas Conditions

**DOI:** 10.3389/fmicb.2017.00954

**Published:** 2017-05-30

**Authors:** Euna Oh, Lynn M. McMullen, Linda Chui, Byeonghwa Jeon

**Affiliations:** ^1^School of Public Health, University of Alberta, EdmontonAB, Canada; ^2^Department of Agricultural, Food and Nutritional Science, University of Alberta, EdmontonAB, Canada; ^3^Department of Laboratory Medicine and Pathology, University of Alberta, EdmontonAB, Canada; ^4^Provincial Laboratory for Public Health, EdmontonAB, Canada

**Keywords:** *Campylobacter*, aerotolerance, virulence genes, bacterial survival, pathogen inhibition

## Abstract

*Campylobacter jejuni* accounts for a significant number of foodborne illnesses around the world. *C. jejuni* is microaerophilic and typically does not survive efficiently in oxygen-rich conditions. We recently reported that hyper-aerotolerant (HAT) *C. jejuni* are highly prevalent in retail poultry meat. To assess the capabilities of HAT *C. jejuni* in foodborne transmission and infection, in this study, we investigated the prevalence of virulence genes in HAT *C. jejuni* and the survival in poultry meat in atmosphere at a refrigeration temperature. When we examined the prevalence of eight virulence genes in 70 *C. jejuni* strains from raw poultry meat, interestingly, the frequencies of detecting virulence genes were significantly higher in HAT *C. jejuni* strains than aerosenstive *C. jejuni* strains. This suggests that HAT *C. jejuni* would potentially be more pathogenic than aerosensitive *C. jejuni*. Under aerobic conditions, aerosensitive *C. jejuni* survived at 4°C in raw poultry meat for 3 days, whereas HAT *C. jejuni* survived in poultry meat for a substantially extended time; there was a five-log CFU reduction over 2 weeks. In addition, we measured the effect of other gas conditions, including N_2_ and CO_2_, on the viability of HAT *C. jejuni* in comparison with aerosensitive and aerotolerant strains. N_2_ marginally affected the viability of *C. jejuni*. However, CO_2_ significantly reduced the viability of *C. jejuni* both in culture media and poultry meat. Based on the results, modified atmosphere packaging using CO_2_ may help us to control poultry contamination with HAT *C. jejuni*.

## Introduction

*Campylobacter* is a leading bacterial cause of human gastroenteritis, annually accounting for approximately 166 million diarrheal cases around the world, particularly in developed countries (Kirk et al., 2015). *Campylobacter* infection in humans develop fever, vomiting, abdominal pains, and diarrhea, and in some cases Guillain–Barré syndrome, an autoimmune disorder characterized by acute and progressive neuromuscular paralysis (Young et al., 2007). Human infection with *C. jejuni* is facilitated by the function of various virulence factors involved in toxin production (e.g., *cdtABC*), cell adhesion (e.g., *cadF*, *peb1A*, and *pldA*) and invasion (e.g., *ciaB*), and colonization of gastrointestinal tracts (Bolton, 2015).

*Campylobacter* is isolated from a wide range of domestic animals and wildlife (Jokinen et al., 2011). In particular, the gastrointestinal tracts of poultry are colonized by *Campylobacter jejuni*, the major human pathogenic species of *Campylobacter*, at the level of 10^6^∼10^8^ CFU/g feces or higher (Hermans et al., 2011). Poultry meat is often contaminated with *C. jejuni* during poultry processing, and human campylobacteriosis is most frequently associated with the consumption of contaminated poultry products (Skarp et al., 2016). In addition, cross-contamination in the kitchen is also an important risk factor transferring *Campylobacter* (Chai et al., 2008; Luber, 2009). It has been estimated that a two-log reduction in the number of *Campylobacter* on chicken carcasses may lead to approximately a 30-fold reduction in the number of human campylobacteriosis cases (Rosenquist et al., 2003). To control *Campylobacter* contamination of poultry, various intervention strategies have been examined at the pre- and post-harvest levels, such as bacteriocin and bacteriophages (Hermans et al., 2011; Umaraw et al., 2017).

Unlike other enteric pathogenic bacteria, *C. jejuni* exhibits unique microbiological features. For example, *C. jejuni* is asaccharolytic and has limitations in the utilization of hexose sugars, including glucose, because of the lack of 6-phosphofructokinase in the glycolysis pathway (Parkhill et al., 2000; Velayudhan and Kelly, 2002). To supply carbon sources, *C. jejuni* relies on the utilization of amino acids, organic acids (e.g., lactic acid), and fucose in some strains (Leach et al., 1997; Thomas et al., 2011; Stahl et al., 2012). In addition, *C. jejuni* is microaerophilic and capnophilic and requires both O_2_ and CO_2_ for growth preferably at 5–10% and 1–10%, respectively (Bolton and Coates, 1983). Despite the fastidious nature of *Campylobacter*, it has not been understood how *Campylobacter* causes such a significant number of human infection cases around the world.

Various tolerance mechanisms have been reported to support the survival of *Campylobacter* under harsh stress conditions, such as heat, cold, acid, and desiccation stresses (Murphy et al., 2006). In addition, *Campylobacter* produces biofilms and switches its physiological state to a viable but nonculturable (VBNC) cell to promote survival under stress conditions. In *C. jejuni*, biofilm formation is stimulated under aerobic conditions, and aeration triggers the formation of VBNC cells (Oh et al., 2015b, 2016), suggesting *C. jejuni* is equipped with multiple survival mechanisms that may support the viability of *C. jejuni* under oxygen-rich conditions. Besides these survival mechanisms, aerotolerance would be the front-line survival mechanism of *C. jejuni* when this microaerophilic pathogen encounters the aerobic environment (Bronowski et al., 2014). Despite our perception about oxygen-sensitivity in *C. jejuni*, interestingly, we recently reported that hyper-aerotolerant (HAT) strains of *C. jejuni* are highly prevalent in retail poultry meat; the HAT strains survive longer than 24 h in vigorous aerobic shaking at 200 rpm. Also, HAT *C. jejuni* often belongs to the multilocus sequence typing (MLST) clonal complexes (CCs) that are frequently implicated in human infection (Oh et al., 2015a), suggesting that HAT *C. jejuni* might be closely related to human infection. To evaluate the virulence potential of HAT *C. jejuni*, in this study, we investigated the prevalence of virulence genes in HAT *C. jejuni* strains. In addition, we measured the survival of HAT *C. jejuni* under different gas conditions, such as N_2_ and CO_2_, aiming to develop intervention strategies to control HAT *C. jejuni* in poultry meat by using modified atmosphere packaging (MAP) with different gases, since aerotolerance confers tolerance to oxygen, not other gases.

## Materials and Methods

### Bacterial Strains and Culture Conditions

Seventy *C. jejuni* strains that were isolated from poultry were used in this study (Oh et al., 2015a). *C. jejuni* NCTC 11168 is the first genome-sequenced strain of *Campylobacter* and was used as a control in the study (Parkhill et al., 2000). *C. jejuni* 81–176 was used as a positive control for PCR detection of *virB11* (Bacon et al., 2002). In our previous study, we first reported high prevalence of HAT *C. jejuni* that can effectively survive in a vigorous aerobic condition, such as aerobic shaking at 200 rpm (Oh et al., 2015a). Based on the level of aerotolerance, we arbitrarily divided *C. jejuni* into three different groups: (1) aerosensitive *C. jejuni* that loses viability before 12 h by aerobic shaking at 200 rpm, (2) aerotolerant *C. jejuni* that loses viability between 12∼24 h by aerobic shaking at 200 rpm, and (3) HAT *C. jejuni* that survives even after 24 h of aerobic shaking at 200 rpm (Oh et al., 2015a). The 70 *C. jejuni* poultry strains were isolated from retail poultry meat in our previous study and consisted of 20 aerosensitive strains, 25 aerotolerant strains, and 25 HAT strains (Oh et al., 2015a). The *C. jejuni* strains were routinely grown on Mueller–Hinton (MH) agar plates (Difco) at 42°C under microaerobic conditions (85% N_2_, 5% O_2_ and 10% CO_2_).

### Determination of *C. jejuni* Survival under Different Gas Conditions

*Campylobacter jejuni* survival was determined in MH media and chicken meat at 4°C in normal atmospheric conditions and under CO_2_ and N_2_. Frozen *C. jejuni* strains in 10% glycerol were inoculated on MH agar plates and inoculated plates were incubated at 42°C under microaerobic condition. Overnight cultures of strains of *C. jejuni* grown on MH agar plates were harvested with fresh MH broth and diluted in MH broth to an optical density at 600 nm (OD_600_) of 0.1. The bacterial suspension was transferred to multiple 96-well plates, and the 96-well plates were incubated at 4°C in air and in an anaerobic jar filled with either CO_2_ or N_2_. In addition, N_2_ gas condition was constructed with 100% nitrogen gas flushing and CO_2_ condition was generated with gas pack (>97% CO_2_). To prevent desiccation, a container with water was placed nearby the 96-well plates in a refrigerator. Samples were taken at predetermined time for enumeration. In addition, the survival of two strains of *C. jejuni*, which were randomly chosen from each aerotolerance group [HAT strains (#12 and #21), aerotolerant strains (#4 and #29), and aerosensitive strains (#24 and #66)], was determined in raw chicken meat; these strains were selected from different MLST CCs based on their aerotolerance level. Approximately one-gram of raw chicken meat, including skin and muscle, was prepared with a sterilized razor and placed in a 12-well plate. After applying an aliquot (100 μl) of *C. jejuni* suspension (approximately 8 × 10^8^ CFU/ml) onto each portion of meat and skin mixture, the plate was stored at 4°C under three different gas conditions, including normal atmosphere, CO_2_, and N_2_. Due to the potential indigenous *C. jejuni* in poultry meat, controls were prepared without addition of *C. jejuni*. The poultry meat samples were transferred to a 50 ml tube containing 2 ml of fresh MH broth. After vortexing for 2 min, the supernatant was collected, serially diluted, and spread onto MH agar plates for enumeration. Each experiment was carried out with duplicate samples, and the experiment was repeated three times.

### PCR Detection of Virulence Genes

Overnight cultures on MH agar at 42°C under microaerobic conditions of *C. jejuni* strains were collected in PBS (pH 7.2). Bacterial suspension of overnight culture of *C. jejuni* strains were diluted in PBS to an OD600 of 0.01 (approximately, 8 × 10^6^ CFU/ml) and boiled for 10 min to release gDNA. After centrifugation, the supernatant was used as a template. To evaluate the potential virulence of HAT *C. jejuni* strains, we investigated the prevalence of eight important virulence genes (*cadF*, *cdtB*, *ciaB*, *docA*, *iam*, *peb1A*, *pldA*, and *virB11*), which are associated with toxin production, cell adhesion and invasion, and colonization of gastrointestinal tracts in chickens with PCR with ExTaq polymerase (Takara, Japan). Primers used are listed in **Table 1**. The positive controls for six virulence genes, such as *cadF*, *cdtB*, *ciaB*, *docA*, *iam*, *peb1A* and *pldA*, were amplified from *C. jejuni* NCTC11168, and *virB11* was amplified from *C. jejuni* 81–176. The PCR mixture was amplified with the following conditions: initial denaturation at 96°C for 3 min followed by 35 cycles of denaturation 96°C for 30 s, variable annealing temperature (*cdtB*, *ciaB*, *cadF* and *pldA* at 45°C, *docA*, *peb1* and *virB11* at 50°C, *iam* at 53°C) for 30 s, extension at 72°C for 1 min 20 s and the final extension at 72°C for 7 min. The results were analyzed by electrophoresis with 1% agarose gels and SYBR safe staining dye (Invitrogen).

**Table 1:** Primers used in this study.

Gene	Primer	Sequence (5′-3′)	Size (bp)	Reference
*cadF*	cadF_F	TTGAAGGTAATTTAGATATG	400	Konkel et al., 1999a
	cadF_R	CTAATACCTAAAGTTGAAAC		
*cdtB*	cdtB_F	GTTAAAATCCCCTGCTATCAACCA	495	Bang et al., 2001
	cdtB_R	GTTGGCACTTGGAATTTGCAAGGC		
*ciaB*	ciaB_F	GTTAAAGTTGGCAGT	1163	Konkel et al., 1999a
	ciaB_R	GTTCTTTAAATTTTTCATAATGC		
*docA*	docA_F	ATAAGGTGCGGTTTTGGC	725	Muller et al., 2006
	docA_R	GTCTTTGCAGTAGATATG		
*iam*	iamA_F	GCACAAAATATATCATTACAA	518	Konkel et al., 1999a
	iamA_R	TTCACGACTACTATGAGG		
*peb1*	peb1_F	TAATACGACTCACTATAGGGGAAAATCTTT	775	Biswas et al., 2011
	peb1_R	TTTTCGCTAAAGCATCAATTTCATT		
*pldA*	pldA_F	AAGCTTATGCGTTTTT	913	Datta et al., 2003
	pldA_R	TATAAGGCTTTCTCCA		
*virB11*	virB11_F	GAACAGGAAGTGGAAAAACTAGC	708	Bacon et al., 2002
	virB11_R	TTCCGCATTGGGCTATATG		

### Statistical Analysis

Two-way ANOVA was performed by using GraphPad Prism 6 (GraphPad Software Inc., United States). Chi-square distribution was used to analyze if the prevalence of virulence genes is dependent on aerotolerance by using SPSS Statistics 21.0 (IBM Predictive Software, United States).

## Results

### Effect of Aerotolerance on *C. jejuni* Survival in Chicken Meat

**FIGURE 1:**
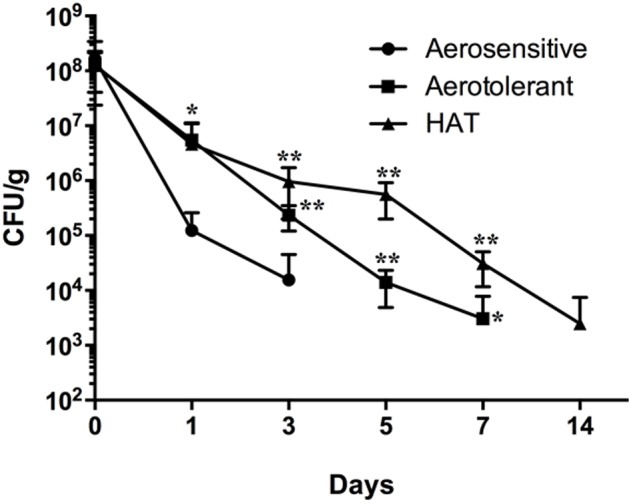
Survival of aerosensitive, aerotolerant, and HAT *C. jejuni* strains in poultry meat at 4°C under aerobic conditions. Two *C. jejuni* strains were randomly selected from each aerotolerance group and used to spike raw poultry meat in duplicate. The results indicate the means and standard deviations of duplicate samples of the two different strains in a single experiment. Three independent experiments were performed, and similar results were obtained in all the experiments. The statistical analysis was performed with two-way ANOVA in comparison with aerosensitive strains. ^∗^*P* ≤ 0.05, ^∗∗^*P* ≤ 0.01.

To evaluate the impact of hyper-aerotolerance on the survival of *C. jejuni* in poultry meat in this study, raw poultry meat was spiked with two strains of *C. jejuni* from each aerotolerance group (i.e., aerosenstive, aerotolerant, and HAT *C. jejuni* groups) and incubated at 4°C under aerobic conditions. The aerosensitive *C. jejuni* strains lost their viability on poultry meat within 3 days, and the aerotolerant *C. jejuni* strains survived for 7 days (**Figure 1**). Interestingly, HAT *C. jejuni* strains survived in poultry meat for 2 weeks (**Figure 1**). This means that HAT *C. jejuni* strains survived in food in atmospheric conditions approximately four times longer than aerosensitive strains of *C. jejuni*. The results showed that aerotolerance significantly affects the viability of *C. jejuni* in poultry meat under aerobic conditions.

### Prevalence of Virulence Genes in HAT *C. jejuni* Strains

**FIGURE 2:**
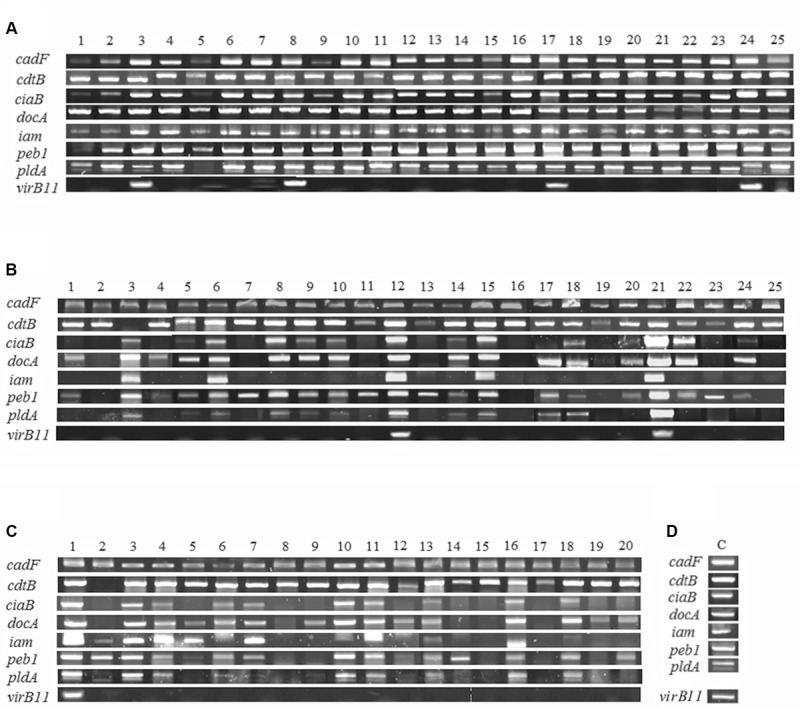
Detection of virulence genes in 70 strains of *C. jejuni* from poultry meat. The results show the prevalence of eight virulence genes in hyper-aerotolerant **(A)**, aerotolerant **(B)**, and aerosensitive **(C)** strains of *C. jejuni*. Positive controls **(D)** were amplified from *C. jejuni* NCTC11168 (*cadF*, *cdtB*, *ciaB*, *docA*, *iam*, *peb1 and pldA*) and 81–176 (*virB11*). Controls were included each batch of PCR testing, and representative results were presented.

In 70 strains of *C. jejuni* from poultry meat, the frequencies of detecting virulence genes were 100, 97.1, 68.6, 81.4, 57.1, 84.3, 64.3, and 11.4% for *cadF*, *cdtB*, *ciaB*, *docA*, *iam*, *peb1*, *pldA*, and *virB11*, respectively (**Figure 2** and **Table 2**). When we clustered the results based on the aerotolerance level, interestingly, the detection frequencies in HAT *C. jejuni* strains were higher than those in aerosensitive *C. jejuni* strains (**Table 2**), suggesting that HAT *C. jejuni* would potentially be more pathogenic to humans than aerosensitive *C. jejuni*.

**Table 2:** Prevalence (%) of virulence genes in 70 isolates of *C. jejuni* from poultry meat.

	*cadF*^ND^	*cdtB*^NS^	*ciaB*^∗∗∗∗^	*docA*^∗∗^	*iam*^∗∗∗∗^	*peb1*^∗^	*pldA*^∗∗∗∗^	*virB11*^NS^
HAT *C. jejuni* (*n* = 25)	100	100	100	100	100	100	96	20
Aerotolerant *C. jejuni* (*n* = 25)	100	96	52	68	20	84	48	8
Aerosensitive *C. jejuni* (*n* = 20)	100	95	50	75	50	65	45	5
Total (*n* = 70)	100	97.1	68.6	81.4	57.1	84.3	64.3	11.4

*Statistical significance was performed by chi-square distribution with SPSS ver.21 (IBM). ^∗^*P* ≤ 0.05, ^∗∗^*P* ≤ 0.01, ^∗∗∗∗^*P* ≤ 0.0001, NS, Not Significant; ND, Not determined*.

### Viability of HAT *C. jejuni* Strains in Different Gas Atmospheres

**FIGURE 3:**
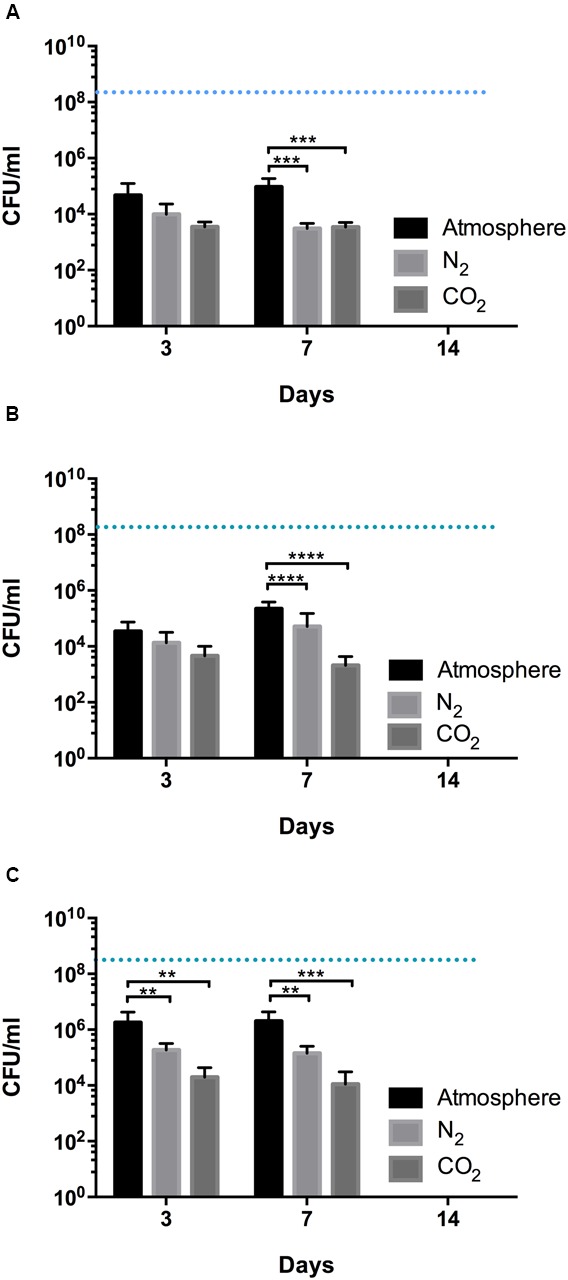
Survival of aerosensitive **(A)**, aerotolerant **(B)**, and HAT **(C)** strains of *C. jejuni* in MH broth under different gas conditions. Incubation was carried out in atmospheric, N_2_, and CO_2_ conditions. Two strains from each aerotolerance group were randomly selected, and each strain was inoculated in MH broth in triplicate. The initial CFU was adjusted to be approximately 10^8^ CFU/ml for all the samples and is indicated with blue dashed lines. The results show the mean and standard deviation of the triplicate samples of two different strains in a single experiment. The experiment was repeated three times, and similar results were obtained in the three independent experiments. Two-way ANOVA testing was carried out for statistical analysis. ^∗∗^*P* ≤ 0.01, ^∗∗∗^*P* ≤ 0.001, ^∗∗∗∗^*P* ≤ 0.0001.

The survival of HAT *C. jejuni* measured under different gaseous conditions. In the food industry, MAP is often employed to extend the microbial shelf-life of meat, and O_2_, N_2_, and CO_2_ are the major gases used for MAP. Thus, we selected N_2_ and CO_2_ for the viability testing of HAT *C. jejuni* strains. Consistent with their aerotolerance level, there was about approximately a four log reduction in CFU in aerosensitive strains of *C. jejuni*, a three log CFU reduction in aerotolerant strains, and a two log CFU reduction in HAT strains of *C. jejuni* at 4°C in MH broth under the normal atmospheric conditions within 3 days. Incubation in N_2_ reduced the survival of *C. jejuni*, and CO_2_ further decreased CFU counts in HAT *C. jejuni*, compared with the aerobic conditions (**Figure 3**). The CFU reduction in all the strains were similar between days 3 and 7 (**Figure 3**), and no *C. jejuni* was detected in day 14 (data not shown).

### Impact of Different Gas Atmosphere on the Survival of HAT *C. jejuni* in Poultry Meat

**FIGURE 4:**
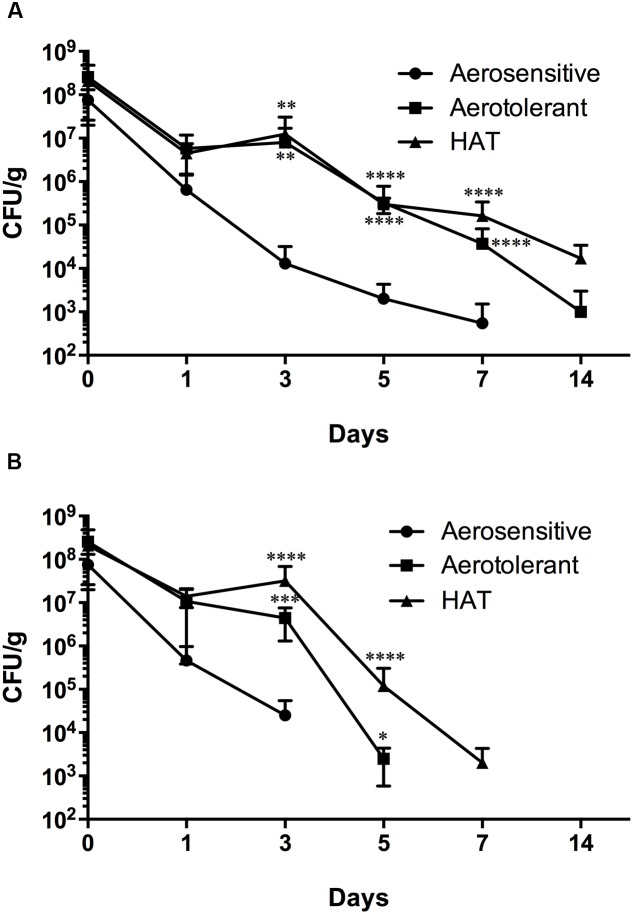
Survival of aerosensitive, aerotolerant, and HAT *C. jejuni* strains at 4°C in poultry meat in N_2_ **(A)** and CO_2_ **(B)**. Two strains from each aerotolerance group were randomly selected for the experiment. The results indicate the means and standard deviations of duplicate samples of the two different strains in a single experiment. Three independent experiments were performed, and similar results were obtained all the experiments. The statistical analysis was carried out with two-way ANOVA. ^∗^*P* ≤ 0.05, ^∗∗^*P* ≤ 0.01, ^∗∗∗^*P* ≤ 0.001, ^∗∗∗∗^*P* ≤ 0.0001.

The viability of *C. jejuni* strains belonging to different aerotolerance groups was determined in poultry meat stored in different gas atmospheres. In N_2_, aerotolerant and HAT *C. jejuni* strains were detected for 14 days, whereas aerosensitive strains survived for 7 days (**Figure 4A**). Compared to aerobic conditions (**Figure 1**), N_2_ did not reduce the viability of HAT *C. jejuni* strains in poultry meat. In CO_2_, however, HAT strains of *C. jejuni* survived only for a week (**Figure 4B**); this is a significant viability reduction compared to atmospheric conditions where HAT *C. jejuni* strains survived for 2 weeks in poultry meat (**Figure 1**). The results exhibit that HAT *C. jejuni* did not survive well in CO_2_, compared to aerobic conditions.

## Discussion

Despite the well-known microaerophilic characteristic of *C. jejuni*, our previous study showed that some *C. jejuni* strains are highly tolerant to aerobic stress and these strains are highly prevalent in poultry meat (Oh et al., 2015a). In addition, Rodrigues et al. (2015) recently characterized an unique human isolate of *C. jejuni* strain, named Bf, which can grow aerobically, suggesting that some *C. jejuni* strains are highly resistant to aerobic stress. Increased tolerance to aerobic stress would enable *C. jejuni* to survive during transmission to humans through foods. This would significantly impact the safety of poultry meat because of frequent contamination of poultry meat by *Campylobacter*. In this study, we demonstrated that HAT *C. jejuni* survived in raw poultry meat at 4°C significantly longer than aerosensitive *C. jejuni* (**Figure 1**), confirming the potential threat of HAT *C. jejuni* on the safety of fresh poultry meat.

The *cadF* and *cdt* genes are detected in *C. jejuni* strains from poultry at high frequencies (Rozynek et al., 2005). Similarly, in this study, *cadF* and *cdt* genes were detected in all and most (97.1%) *C. jejuni* strains, respectively (**Table 2**). The *iam* locus has been detected in *C. jejuni* chicken isolates at 54.7% (Rozynek et al., 2005). The *pldA* and *ciaB* genes have been detected from *C. jejuni* poultry isolates at the frequencies of 63.6 and 67.3%, respectively (Melo et al., 2013). Hanning et al. (2010) reported relatively low detection frequencies of *ciaB* (40%) and *pldA* (56%) in *C. jejuni* isolates from poultry carcasses. The *virB11* gene is located in the virulence plasmid pVir, which is often detected in *C. jejuni* strains that cause bloody diarrhea (Bacon et al., 2002; Tracz et al., 2005). The prevalence of *virB11* was 10.7∼17% in human clinical isolates and 9.5∼14% in poultry isolates (Datta et al., 2003; Tracz et al., 2005). When the results were sorted based on the aerotolerance level, the frequencies of detecting virulence genes were significantly higher in HAT *C. jejuni* strains in comparison with aerosenstive *C. jejuni* strains (**Figure 2** and **Table 2**). Interestingly, the most substantial differences in the frequency of detection were observed in the genes associated with invasion, including *ciaB* and *iam* (**Figure 2** and **Table 2**). CiaB shares similarities with SipB (*Salmonella* invasion protein B) from *Salmonella* and IpaB (invasion plasmid antigen B) from *Shigella flexneri* and is translocated to human epithelial cells. Even though a knockout mutation of *ciaB* does not affect *C. jejuni* adhesion to INT407 cells, it significantly impairs the internalization of *C. jejuni* into INT407 cells (Konkel et al., 1999b). The invasion-associated marker (*iam*) locus was first reported by Carvalho et al. (2001) with random amplified polymorphic DNA techniques (RAPD) and was detected in 85% of invasive strains and 20% of non-invasive strains. The detection frequencies of *pldA* were also significantly different between HAT and aerosensitive *C. jejuni* strains (**Figure 2** and **Table 2**). The *pldA* gene encodes an outer membrane phospholipase A that is involved in hemolysis (Grant et al., 1997). The *pldA* and *ciaB* genes also play a role in *C. jejuni* colonization of chicken intestines (Ziprin et al., 2001). The increased prevalence of the virulence genes in HAT *C. jejuni* strains suggests that HAT *C. jejuni* would be more pathogenic to humans than aerosensitive *C. jejuni*.

The transmission of *C. jejuni* to humans is primarily mediated by contaminated food, mainly poultry meat. Due to the fastidiousness and oxygen sensitivity, *C. jejuni* is not expected to survive efficiently during foodborne transmission in oxygen-rich, atmospheric conditions. However, our results indicate that HAT *C. jejuni* survives longer in poultry meat than aerosensitive strains during transmission to humans in air and would be more capable of causing human infection (**Figure 1**). In this study, we observed that the survival of HAT *C. jejuni* is significantly reduced under CO_2_ (**Figures 3**, **4**). This provides important scientific background for developing methods to control HAT *C. jejuni* with MAP. In food industry, CO_2_, N_2_ and their combinations are generally used for the development of MAP of foods. Compared to aerobic conditions, the survival of HAT *C. jejuni* strains in raw poultry meat was significantly reduced by CO_2_ (**Figures 1**, **4B**). Meredith et al. (2014) tested different compositions of the three gases and reported that 40:30:30 of CO_2_:O_2_:N_2_ is the optimum gas mixture both to reduce *Campylobacter* and to extend shelf-life in poultry filets. The threshold CO_2_ concentration that critically affects the viability of HAT *C. jejuni* has not been examined, and its determination still awaits future studies for the development of optimal gas mixtures of MAP to control HAT *C. jejuni* in poultry meat.

Our previous study revealed that most HAT *C. jejuni* strains belong to MLST CC 21 (Oh et al., 2015a), the major MLST CC implicated in human gastroenteritis (Nielsen et al., 2010). It is possible that strains of *C. jejuni* with increased aerotolerance may survive well in foods and are more likely to reach humans, consequently causing human illnesses more frequently than aerosensitive *C. jejuni* strains. At this stage, it remains unknown why HAT *C. jejuni* strains harbor more virulence genes than oxygen-sensitive strains. In this study, we did not provide empirical evidences about the virulence, such as invasion of and adhesion to epithelial cells, and such works will be done in future studies. Nevertheless, this study also showed that MAP using CO_2_ may be an interesting approach to control HAT *C. jejuni* in poultry meat.

## Author Contributions

Design of the project: EO and BJ. Performance of the experiments: EO. Data analysis: EO, LM, LC, and BJ. Writing of the manuscript: EO, LM, LC, and BJ.

## Conflict of Interest Statement

The authors declare that the research was conducted in the absence of any commercial or financial relationships that could be construed as a potential conflict of interest.
